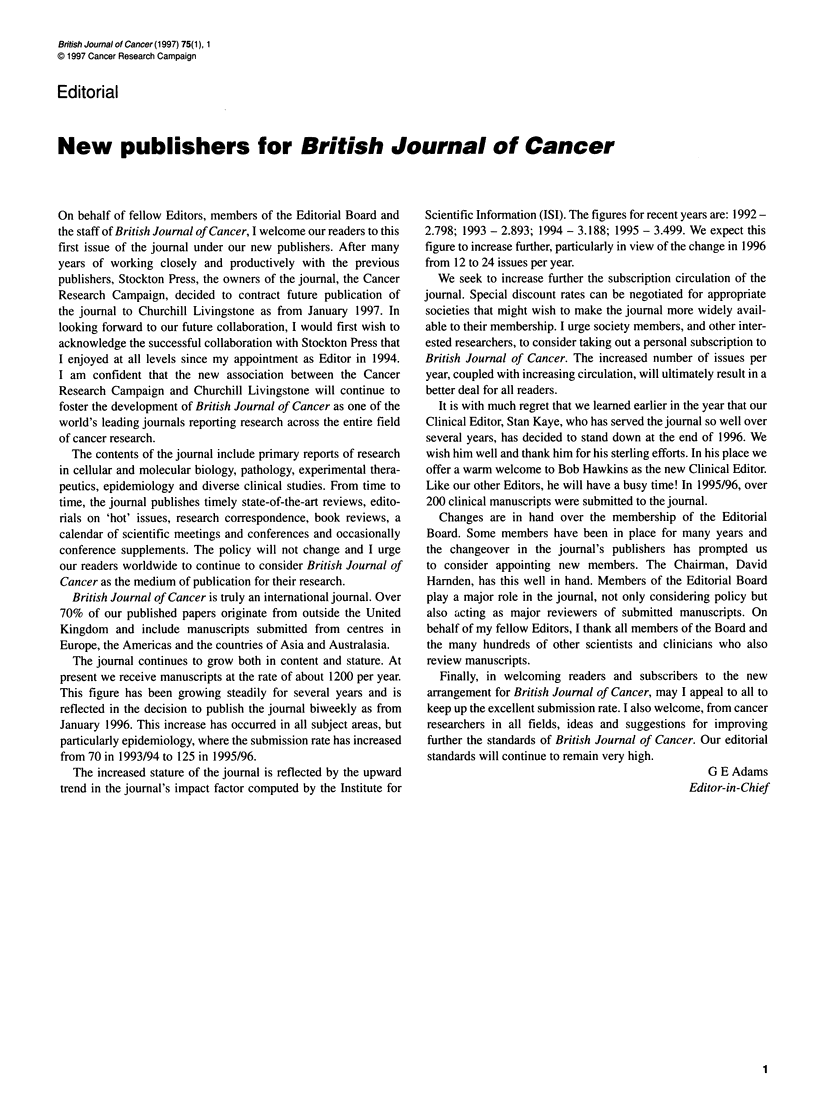# New publisher for British Journal of Cancer

**Published:** 1997

**Authors:** G E Adams


*British Journal of Cancer* (1997) **75**(1), 1
© 1997 Cancer Research Campaign

Editorial

### New publishers for *British Journal of Cancer*

On behalf of fellow Editors, members of the Editorial Board and the staff of *British Journal of Cancer*, I welcome our readers to this first issue of the journal under our new publishers. After many years of working closely and productively with the previous publishers, Stockton Press, the owners of the journal, the Cancer Research Campaign, decided to contract future publication of the journal to Churchill Livingstone as from January 1997. In looking forward to our future collaboration, I would first wish to acknowledge the successful collaboration with Stockton Press that I enjoyed at all levels since my appointment as Editor in 1994. I am confident that the new association between the Cancer Research Campaign and Churchill Livingstone will continue to foster the development of *British Journal of Cancer* as one of the world's leading journals reporting research across the entire field of cancer research.

The contents of the journal include primary reports of research in cellular and molecular biology, pathology, experimental therapeutics, epidemiology and diverse clinical studies. From time to time, the journal publishes timely state-of-the-art reviews, editorials on 'hot' issues, research correspondence, book reviews, a calendar of scientific meetings and conferences and occasionally conference supplements. The policy will not change and I urge our readers worldwide to continue to consider *British Journal of Cancer* as the medium of publication for their research.

*British Journal of Cancer* is truly an international journal. Over 70% of our published papers originate from outside the United Kingdom and include manuscripts submitted from centres in Europe, the Americas and the countries of Asia and Australasia.

The journal continues to grow both in content and stature. At present we receive manuscripts at the rate of about 1200 per year. This figure has been growing steadily for several years and is reflected in the decision to publish the journal biweekly as from January 1996. This increase has occurred in all subject areas, but particularly epidemiology, where the submission rate has increased from 70 in 1993/94 to 125 in 1995/96.

The increased stature of the journal is reflected by the upward trend in the journal's impact factor computed by the Institute for Scientific Information (ISI). The figures for recent years are: 1992 – 2.798; 1993 – 2.893; 1994 – 3.188; 1995 – 3.499. We expect this figure to increase further, particularly in view of the change in 1996 from 12 to 24 issues per year.

We seek to increase further the subscription circulation of the journal. Special discount rates can be negotiated for appropriate societies that might wish to make the journal more widely available to their membership. I urge society members, and other interested researchers, to consider taking out a personal subscription to *British Journal of Cancer*. The increased number of issues per year, coupled with increasing circulation, will ultimately result in a better deal for all readers.

It is with much regret that we learned earlier in the year that our Clinical Editor, Stan Kaye, who has served the journal so well over several years, has decided to stand down at the end of 1996. We wish him well and thank him for his sterling efforts. In his place we offer a warm welcome to Bob Hawkins as the new Clinical Editor. Like our other Editors, he will have a busy time! In 1995/96, over 200 clinical manuscripts were submitted to the journal.

Changes are in hand over the membership of the Editorial Board. Some members have been in place for many years and the changeover in the journal's publishers has prompted us to consider appointing new members. The Chairman, David Harnden, has this well in hand. Members of the Editorial Board play a major role in the journal, not only considering policy but also acting as major reviewers of submitted manuscripts. On behalf of my fellow Editors, I thank all members of the Board and the many hundreds of other scientists and clinicians who also review manuscripts.

Finally, in welcoming readers and subscribers to the new arrangement for *British Journal of Cancer*, may I appeal to all to keep up the excellent submission rate. I also welcome, from cancer researchers in all fields, ideas and suggestions for improving further the standards of *British Journal of Cancer*. Our editorial standards will continue to remain very high.

G E Adams
*Editor-in-Chief*